# Development and validation of prognostic nomograms for early-onset locally advanced colon cancer

**DOI:** 10.18632/aging.202157

**Published:** 2020-12-03

**Authors:** Yuqiang Li, Wenxue Liu, Zhongyi Zhou, Heming Ge, Lilan Zhao, Heli Liu, Xiangping Song, Dan Wang, Qian Pei, Fengbo Tan

**Affiliations:** 1 Department of Gastrointestinal Surgery, Xiangya Hospital, Central South University, Changsha, China; 2 Department of Rheumatology, Guangdong Provincial People's Hospital, Guangdong Academy of Medical Sciences, Guangzhou, China; 3 Department of Cardiology, Xiangya Hospital, Central South University, Changsha, China; 4 Department of Thoracic Surgery, Fujian Provincial Hospital, Fuzhou, China

**Keywords:** early-onset colon cancer, locally advanced colon cancer, overall survival, cancer-specific survival, nomogram

## Abstract

Background: The incidence of colorectal cancer in patients younger than 50 years has been increasing in recent years.

Objective: Develop and validate prognostic nomograms predicting overall survival (OS) and cancer-specific survival (CSS) for early-onset locally advanced colon cancer (EOLACC) based on the Surveillance, Epidemiology, and End Results (SEER) database.

Results: The entire cohort comprised 13,755 patients with EOLACC. The nomogram predicting OS for EOLACC displayed that T stage contributed the most to prognosis, followed by N stage, regional nodes examined (RNE) and surgery. The nomogram predicting CSS for EOLACC demonstrated similar results. Various methods identified the discriminating superiority of the nomograms. X-tile software was used to classify patients into high-risk, medium-risk, and low-risk according to the risk score of the nomograms. The risk stratification effectively avoided the survival paradox.

Conclusions: We established and validated nomograms for predicting OS and CSS based on a national cohort of almost 13,000 EOLACC patients. The nomograms could effectively solve the issue of survival paradox of the AJCC staging system and be an excellent tool to integrate the clinical characteristics to guide the therapeutic choice for EOLACC patients.

Methods: Nomograms were constructed based on the SEER database and the Cox regression model.

## INTRODUCTION

Colorectal cancer ranks as the fourth most frequently diagnosed cancer and the second leading cause of cancer death in the United States [1]. The incidence and mortality rates of colorectal cancer have decreased in recent years thanks to the popularity of colonoscopy screening and the advancement of treatment [2]. However, the incidence of colorectal cancer in patients younger than 50 years has been increasing [3]. Recent news that Chadwick Aaron Boseman died due to colon cancer at the age of 43 has aroused worldwide concern for early-onset colon cancer. Furthermore, it is difficult to advise treatment options for early-onset colorectal cancer because the prognosis of colon cancer among young patients is not well known [4]. Therefore, early-onset colorectal cancer should gain more attention.

Colon cancer accounts for the vast majority of colorectal cancer, around 70% [3, 5, 6]. Although colorectal cancer is usually discussed as a general category, there are many differences, involving embryological origin, anatomy, function as well as treatments, between colon cancer and rectal cancer [7]. In addition, numerous studies tend to put patients with stage II and stage III colon cancer together in exploring prognostic information [8, 9] due to the relatively consistent treatment strategies and follow-up principles. Therefore, this study focused on locally advanced colon cancer patients younger than 50 years (early-onset locally advanced colon cancer; EOLACC).

The Surveillance, Epidemiology, and End Results (SEER) program of the National Cancer Institute (NCI) is a source for epidemiologic information on the incidence and survival rates of cancer in the United States [10]. Various studies have explored clinical problems by analyzing the data from the SEER database, which has helped to further improve the treatment of cancer patients. Although widely used to evaluate the prognosis of various tumors, the American Joint Committee on Cancer (AJCC) staging system contains a survival paradox for locally advanced colon cancer, that colon cancer patients with T3-4N0 (stage II) had an approximate or even worse survival rate compared to those with T1-2N+ (stage III) [11–13]. The shortcomings of AJCC staging for colon cancer prompted the exploration of a new risk scoring system. The nomogram is widely applied to predict outcomes intuitively and effectively in medical studies. The length of the line in the nomogram can be used to indicate the impact of each variable on the outcome.

Therefore, our plan was to develop and validate prognostic nomograms predicting overall survival (OS) and cancer-specific survival (CSS) for early-onset locally advanced colon cancer based on the SEER database.

## RESULTS

### Patient characteristics

The entire cohort from the SEER database comprised 13,755 patients with histologically confirmed locally advanced colon cancer, who were younger than 50-year-old; these were distributed into a training group or a verification group randomly according to the ratio of 2:1. Table 1 summarizes the demographic, clinical as well as pathological characteristics of the study cohort. The target population was mainly 40-49 years old (74.30%) and White (72.32%). Male patients had a slightly predominance compared with females (52.05% vs. 47.95%) in EOLACC. Meanwhile, early-onset patients with locally advanced left colon cancer were slightly more than those with locally advanced right colon cancer (50.69% vs. 47.29%). Moreover, mucinous cell carcinoma (MCC)/signet ring cell carcinoma (SRCC) accounted for 13.41% of cases in this study. Besides, the proportion of stage III colon cancer (N+: N1 and N2) was higher than that of stage II (N0) (57.72% vs. 42.28%). Almost all of the patients (99.00%) had undergone colectomy and 64.18% of them received chemotherapy. More importantly, the patients with RNE ≥ 12 totaled 86.75%. In addition, 3.56% received radiotherapy, which is not a conventional treatment for colon cancer.

**Table 1:** Characteristics of patients with EOLACC in the training and validation group.

**Characteristics**	**Total(n=13755)**		**Training group(n=9170)**		**Validation group(n=4585)**	
	**N**	**%**	**N**	**%**	**N**	**%**
Gender						
Female	6596	47.95%	4431	48.32%	2165	47.22%
Male	7159	52.05%	4739	51.68%	2420	52.78%
Age(years)						
18-29	633	4.60%	441	4.81%	192	4.19%
30-39	2902	21.10%	1886	20.57%	1016	22.16%
40-49	10220	74.30%	6843	74.62%	3377	73.65%
Marital status						
Married	7815	56.82%	5248	57.23%	2567	55.99%
Unmarried/NOS	5940	43.18%	3922	42.77%	2018	44.01%
Race						
White	9947	72.32%	6636	72.37%	3311	72.21%
Black	2218	16.13%	1466	15.99%	752	16.40%
Other/NOS	1590	11.56%	1068	11.65%	522	11.38%
Tumor location						
Right colon	6505	47.29%	4305	46.95%	2200	47.98%
Left colon	6973	50.69%	4665	50.87%	2308	50.34%
NOS	277	2.01%	200	2.18%	77	1.68%
Pathological grade						
I	835	6.07%	567	6.18%	268	5.85%
II	9616	69.91%	6419	70.00%	3197	69.73%
III	2569	18.68%	1711	18.66%	858	18.71%
IV	434	3.16%	274	2.99%	160	3.49%
NOS	301	2.19%	199	2.17%	102	2.22%
Histological type						
Adenocarcinomas	11911	86.59%	7939	86.58%	3972	86.63%
MCC/SRCC	1844	13.41%	1231	13.42%	613	13.37%
T stage						
T1-2	946	6.88%	627	6.84%	319	6.96%
T3	9997	72.68%	6644	72.45%	3353	73.13%
T4a	1618	11.76%	1102	12.02%	516	11.25%
T4b	1194	8.68%	797	8.69%	397	8.66%
N stage						
N0	5816	42.28%	3889	42.41%	1927	42.03%
N1	4800	34.90%	3175	34.62%	1625	35.44%
N2	3139	22.82%	2106	22.97%	1033	22.53%
Surgery						
Colectomy	13618	99.00%	9075	98.96%	4543	99.08%
Non-colectomy/NOS	137	1.00%	95	1.04%	42	0.92%
Radiotherapy						
Yes	489	3.56%	313	3.41%	176	3.84%
No/Unknown	13266	96.44%	8857	96.59%	4409	96.16%
Chemotherapy						
Yes	8828	64.18%	5872	64.03%	2956	64.47%
No/Unknown	4927	35.82%	3298	35.97%	1629	35.53%
RNE						
<6	450	3.27%	306	3.34%	144	3.14%
6-11	1286	9.35%	877	9.56%	409	8.92%
12-17	3676	26.72%	2455	26.77%	1221	26.63%
18-23	3154	22.93%	2083	22.72%	1071	23.36%
24-39	2062	14.99%	1363	14.86%	699	15.25%
30-35	1194	8.68%	820	8.94%	374	8.16%
≥36	1846	13.42%	1208	13.17%	638	13.91%
NOS	87	0.63%	58	0.63%	29	0.63%
Tumor size						
≤5cm	7031	51.12%	4722	51.49%	2309	50.36%
5-10cm	5484	39.87%	3631	39.60%	1853	40.41%
>10cm	632	4.59%	414	4.51%	218	4.75%
NOS	608	4.42%	403	4.39%	205	4.47%
CEA						
Normal	5706	41.48%	3804	41.48%	1902	41.48%
Elevated	2997	21.79%	1959	21.36%	1038	22.64%
NOS	5052	36.73%	3407	37.15%	1645	35.88%
OS (months)	54 (24-96)		53 (24-96)		55 (24-97)	
CSS (months)	54 (24-97)		54 (24-96)		55 (24-97)	

MCC: mucinous cell carcinoma; SRCC: signet ring cell carcinoma; RNE: regional nodes examined; NOS: Not Otherwise Specified.

Table 2 summarizes the characteristics of EOLACC patients from the external verification group, which comprised 126 patients from China. All of the patients in the external verification group had undergone colectomy. And 66.67% of those from our institute received chemotherapy.

**Table 2:** Characteristics of patients with EOLACC in the external verification group.

**Characteristics**	**External verification group (n=126)**	
	**N**	**%**
Gender		
Female	61	48.41%
Male	65	51.59%
Age(years)		
18-29	8	6.35%
30-39	20	15.87%
40-49	98	77.78%
Marital status		
Married	103	81.75%
Unmarried/NOS	23	18.25%
Race		
White	0	0.00%
Black	0	0.00%
Other/NOS	126	100.00%
Tumor location		
Right colon	66	52.38%
Left colon	60	47.62%
NOS	0	0.00%
Pathological grade		
I	7	5.56%
II	82	65.08%
III	33	26.19%
IV	4	3.17%
NOS	0	0.00%
Histological type		
Adenocarcinomas	101	80.16%
MCC/SRCC	25	19.84%
T stage		
T1-2	4	3.17%
T3	72	57.14%
T4a	31	24.60%
T4b	19	15.08%
N stage		
N0	17	13.49%
N1	63	50.00%
N2	46	36.51%
Surgery		
Colectomy	126	100.00%
Non-colectomy/NOS	0	0.00%
Radiotherapy		
Yes	1	0.79%
No/Unknown	125	99.21%
Chemotherapy		
Yes	84	66.67%
No/Unknown	42	33.33%
RNE		
<6	1	0.79%
6-11	22	17.46%
12-17	56	44.44%
18-23	23	18.25%
24-39	19	15.08%
30-35	3	2.38%
≥36	2	1.59%
NOS	0	0.00%
Tumor size		
≤5cm	88	69.84%
5-10cm	35	27.78%
>10cm	1	0.79%
NOS	2	1.59%
CEA		
Normal	78	61.90%
Elevated	46	36.51%
NOS	2	1.59%
OS (months)	56(27-85)
CSS (months)	54(27-89)

MCC: mucinous cell carcinoma; SRCC: signet ring cell carcinoma; RNE: regional nodes examined; NOS: Not Otherwise Specified.

### Screening independent prognostic factors

The independent prognostic factors affecting OS and CSS were differentiated by univariable and multivariable Cox regression models. The qualified factors in the univariate analysis were brought into the Cox regression model for multivariate analysis. OS was significantly associated with 10 features, including marital status, race, gender, pathological grade, T stage, N stage, surgery, chemotherapy, RNE and carcinoembryonic antigen (CEA) (Table 3). CSS was related to 9 variables (i.e. marital status, race, pathological grade, T stage, N stage, surgery, chemotherapy, RNE and CEA) (Table 4).

**Table 3:** Univariable and multivariable cox regression model analyses of OS for nomogram.

**Characteristics**	**Univariable analysis**				**Multivariable analysis**			
	**OR**	**95% CI lower**	**95% CI upper**	**p-value**	**OR**	**95% CI lower**	**95% CI upper**	**p-value**
Gender				0.004				0.010
Female		reference				reference		
Male	1.140	1.042	1.248	0.004	1.127	1.028	1.235	0.010
Age(years)				0.322				
18-29		reference				NA		
30-39	0.948	0.749	1.199	0.654				
40-49	1.035	0.833	1.287	0.756				
Marital status				<0.001				<0.001
Married		reference				reference		
Unmarried/NOS	1.523	1.392	1.667	<0.001	1.334	1.217	1.463	<0.001
Race				<0.001				<0.001
White		reference				reference		
Black	1.462	1.305	1.638	<0.001	1.436	1.278	1.613	<0.001
Other/NOS	0.939	0.808	1.093	0.417	0.923	0.793	1.074	0.300
Tumor location				.151				
Right colon		reference				NA		
Left colon	0.950	0.867	1.040	0.267				
NOS	1.225	0.921	1.628	0.163				
Pathological grade				<0.001				<0.001
I		reference				reference		
II	1.171	0.946	1.449	0.146	1.071	0.863	1.328	0.534
III	1.856	1.484	2.322	<0.001	1.339	1.067	1.681	0.012
IV	2.094	1.525	2.875	<0.001	1.415	1.027	1.949	0.034
NOS	2.038	1.473	2.819	<0.001	1.260	0.905	1.754	0.171
Histological type				<0.001				.059
Adenocarcinomas		reference				reference		
MCC/SRCC	1.313	1.165	1.481	<0.001	1.127	0.996	1.276	0.059
T stage				<0.001				<0.001
T1-2		reference				reference		
T3	1.444	1.155	1.807	0.001	1.787	1.420	2.248	<0.001
T4a	3.142	2.467	4.001	<0.001	3.177	2.480	4.070	<0.001
T4b	3.857	3.019	4.927	<0.001	3.979	3.083	5.135	<0.001
N stage				<0.001				<0.001
N0		reference				reference		
N1	1.502	1.337	1.687	<0.001	1.735	1.532	1.964	<0.001
N2	3.102	2.775	3.468	<0.001	3.521	3.112	3.983	<0.001
Surgery				<0.001				<0.001
Colectomy		reference				reference		
Non-colectomy/NOS	3.522	2.593	4.782	<0.001	2.426	1.724	3.414	<0.001
Radiotherapy				<0.001				0.228
Yes		reference				reference		
No/Unknown	0.585	0.481	0.711	<0.001	.882	0.720	1.082	0.228
Chemotherapy				<0.001				<0.001
Yes		reference				reference		
No/Unknown	0.836	0.759	0.921	<0.001	1.242	1.118	1.380	<0.001
RNE				<0.001				<0.001
<6		reference				reference		
6-11	0.638	0.516	0.789	<0.001	0.638	0.507	0.802	<0.001
12-17	0.527	0.433	0.641	<0.001	0.496	0.400	0.615	<0.001
18-23	0.480	0.392	0.587	<0.001	0.438	0.351	0.546	<0.001
24-39	0.411	0.329	0.512	<0.001	0.375	0.296	0.476	<0.001
30-35	0.396	0.309	0.507	<0.001	0.355	0.273	0.462	<0.001
≥36	0.455	0.365	0.567	<0.001	0.427	0.336	0.541	<0.001
NOS	0.998	0.654	1.523	0.992	0.700	0.454	1.078	0.106
Tumor size				.267				
≤5cm		reference				NA		
5-10cm	1.024	0.931	1.128	0.622				
>10cm	1.187	0.958	1.472	0.118				
NOS	1.150	0.941	1.407	0.173				
CEA				<0.001				<0.001
Normal		reference				reference		
Elevated	1.873	1.669	2.101	<0.001	1.582	1.407	1.778	<0.001
NOS	1.271	1.142	1.414	<0.001	1.225	1.099	1.365	<0.001

MCC: mucinous cell carcinoma; SRCC: signet ring cell carcinoma; RNE: regional nodes examined; NOS: Not Otherwise Specified; NA: Unavailable.

**Table 4:** Univariable and multivariable cox regression model analyses of CSS for nomogram.

**Characteristics**	**Univariable analysis**				**Multivariable analysis**			
	**OR**	**95% CI lower**	**95% CI upper**	**p-value**	**OR**	**95% CI lower**	**95% CI upper**	**p-value**
Gender				0.095				
Female		reference				NA		
Male	1.093	0.985	1.212	0.095				
Age(years)				0.699				
18-29		reference				NA		
30-39	0.896	0.695	1.155	0.398				
40-49	0.920	0.728	1.162	0.484				
Marital status				<0.001				<0.001
Married		reference				reference		
Unmarried/NOS	1.488	1.341	1.650	<0.001	1.284	1.155	1.428	<0.001
Race				<0.001				<0.001
White		reference				reference		
Black	1.532	1.347	1.743	<0.001	1.526	1.337	1.743	<0.001
Other/NOS	1.010	0.853	1.196	0.911	0.991	0.836	1.174	0.913
Tumor location				0.260				
Right colon		reference				NA		
Left colon	0.952	0.857	1.057	0.355				
NOS	1.217	0.878	1.688	0.238				
Pathological grade				<0.001				0.002
I		reference				reference		
II	1.191	0.927	1.529	0.171	1.045	0.812	1.346	0.732
III	2.009	1.547	2.610	<0.001	1.323	1.014	1.727	0.039
IV	2.413	1.693	3.440	<0.001	1.432	1.000	2.051	0.050
NOS	2.142	1.463	3.136	<0.001	1.167	0.789	1.726	0.439
Histological type				<0.001				0.079
Adenocarcinomas		reference				reference		
MCC/SRCC	1.356	1.183	1.555	<0.001	1.137	0.985	1.313	0.079
T stage				<0.001				<0.001
T1-2		reference				reference		
T3	1.457	1.109	1.914	0.007	1.861	1.407	2.462	<0.001
T4a	3.630	2.717	4.850	<0.001	3.647	2.709	4.910	<0.001
T4b	4.652	3.475	6.229	<0.001	4.986	3.665	6.782	<0.001
N stage				<0.001				<0.001
N0		reference				reference		
N1	1.885	1.640	2.168	<0.001	2.132	1.839	2.473	<0.001
N2	4.029	3.525	4.605	<0.001	4.435	3.826	5.141	<0.001
Surgery				<0.001				<0.001
Colectomy		reference				reference		
Non-colectomy/NOS	3.732	2.642	5.271	<0.001	2.846	1.915	4.228	<0.001
Radiotherapy				<0.001				0.431
Yes		reference				reference		
No/Unknown	0.566	0.455	0.705	<0.001	0.912	0.725	1.147	0.431
Chemotherapy				<0.001				0.034
Yes		reference				reference		
No/Unknown	0.708	0.631	0.795	<0.001	1.144	1.010	1.296	0.034
RNE				<0.001				<0.001
<6		reference				reference		
6-11	0.660	0.517	0.842	0.001	0.637	0.488	0.830	0.001
12-17	0.512	0.408	0.642	<0.001	0.464	0.361	0.597	<0.001
18-23	0.496	0.393	0.625	<0.001	0.431	0.333	0.558	<0.001
24-39	0.407	0.315	0.524	<0.001	0.352	0.266	0.465	<0.001
30-35	0.362	0.271	0.484	<0.001	0.312	0.228	0.427	<0.001
≥36	0.429	0.332	0.555	<0.001	0.384	0.290	0.510	<0.001
NOS	1.036	0.645	1.664	0.883	0.695	0.427	1.131	0.143
Tumor size				0.011				0.352
≤5cm		reference				reference		
5-10cm	1.060	0.949	1.184	0.304	1.052	0.936	1.183	0.394
>10cm	1.424	1.133	1.790	0.002	0.996	0.778	1.276	0.976
NOS	1.220	0.965	1.541	0.096	0.840	0.655	1.077	0.169
CEA				<0.001				<0.001
Normal		reference				reference		
Elevated	1.932	1.692	2.205	<0.001	1.551	1.354	1.775	<0.001
NOS	1.327	1.173	1.502	<0.001	1.277	1.127	1.448	<0.001

MCC: mucinous cell carcinoma; SRCC: signet ring cell carcinoma; RNE: regional nodes examined; NOS: Not Otherwise Specified; NA: Unavailable.

### Development and verification of prognostic nomograms

Based on the results of the multivariable Cox regression models, the nomograms predicting 3-, 5- and 10-year OS and CSS were created with the independent prognostic factors. By adding up the scores related to each variable and projecting total scores to the bottom scales, it is easy to calculate the estimated 3-, 5-, and 10-year OS and CSS probabilities.

**Figure 1:**
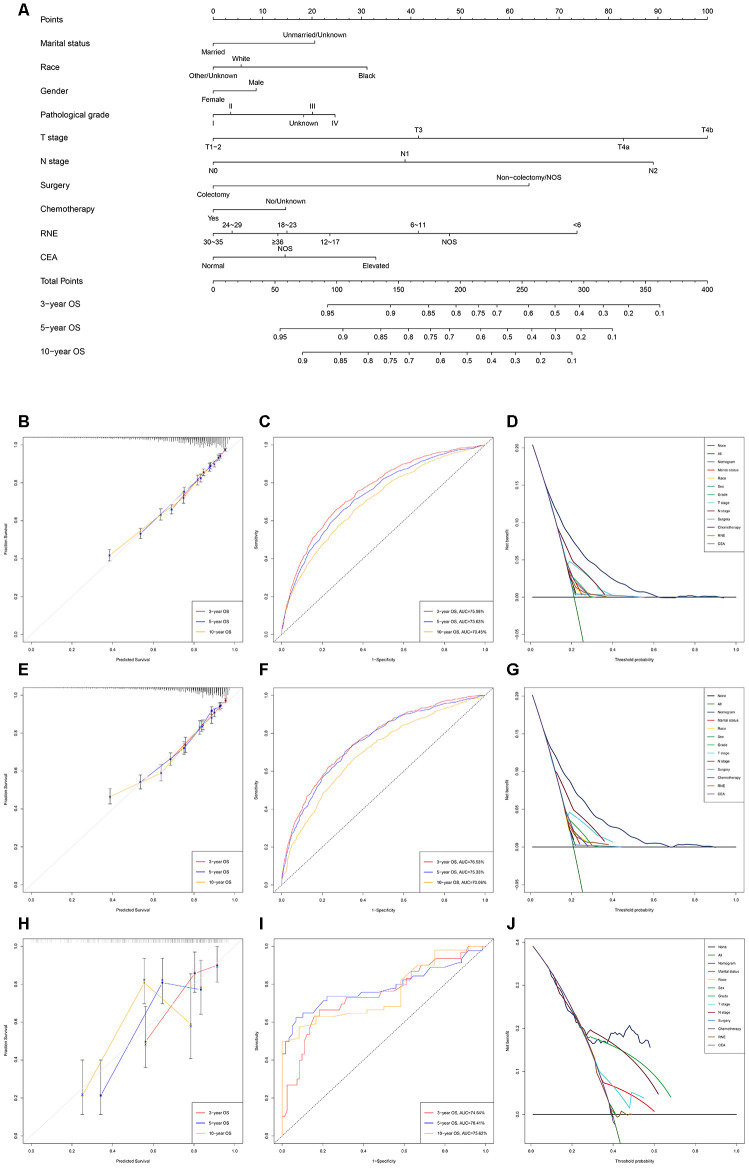
**Development and verification of the nomogram predicting OS.** (**A**) The nomogram of predicting OS for patients with EOLACC. (**B**) The calibration curves predicting OS in the training group. (**C**) The time-dependent ROC curves of nomogram predicting OS in the training group. (**D**) The decision curve analysis of the nomogram and all prognostic factors for OS in the training cohort. (**E**) The calibration curves predicting OS in the verification group. (**F**) The time-dependent ROC curves of nomogram predicting OS in the verification group. (**G**) The decision curve analysis of the nomogram and all prognostic factors for OS in the verification. (**H**) The calibration curves predicting OS in the external verification group. (**I**) The time-dependent ROC curves of nomogram predicting OS in the external verification group. (**J**) The decision curve analysis of the nomogram and all prognostic factors for OS in the external verification.

**Figure 2:**
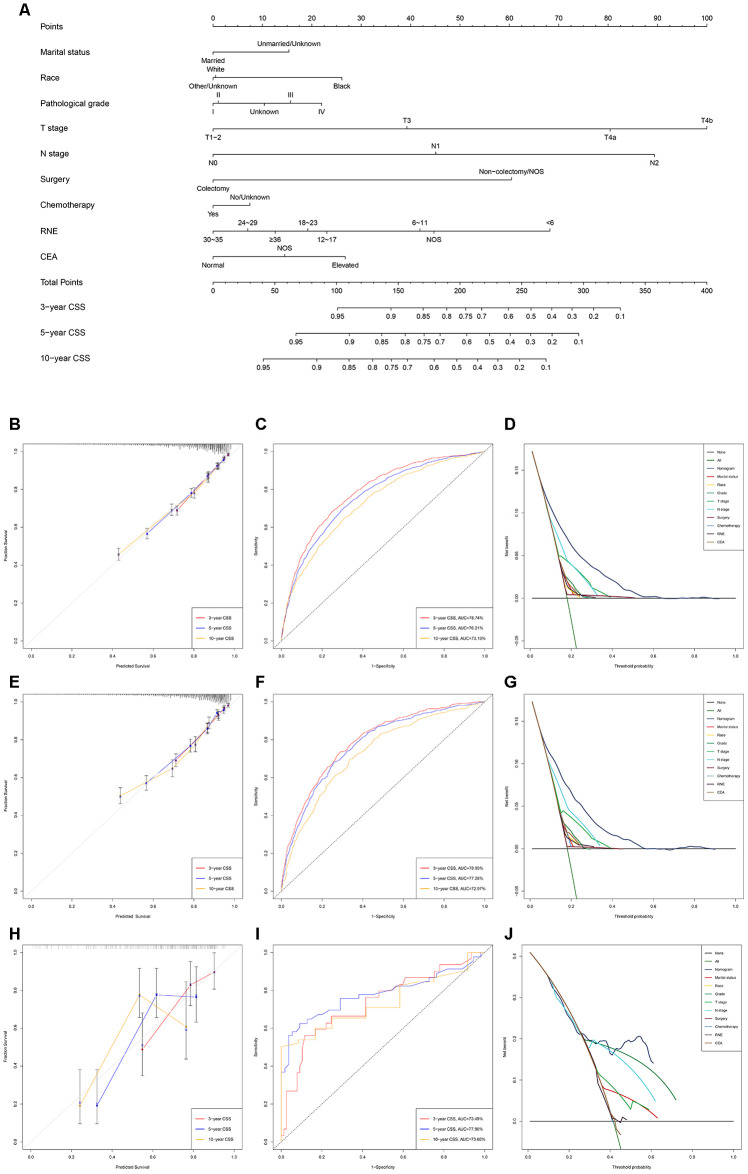
**Development and verification of the nomogram predicting CSS.** (**A**) The nomogram of predicting CSS for patients with EOLACC. (**B**) The calibration curves predicting CSS in the training group. (**C**) The time-dependent ROC curves of nomogram predicting CSS in the training group. (**D**) The decision curve analysis of the nomogram and all prognostic factors for CSS in the training cohort. (**E**) The calibration curves predicting CSS in the verification group. (**F**) The time-dependent ROC curves of nomogram predicting CSS in the verification group. (**G**) The decision curve analysis of the nomogram and all prognostic factors for CSS in the verification. (**H**) The calibration curves predicting CSS in the external verification group. (**I**) The time-dependent ROC curves of nomogram predicting CSS in the external verification group. (**J**) The decision curve analysis of the nomogram and all prognostic factors for CSS in the external verification.

The nomogram predicting OS and CSS for EOLACC displayed that T stage contributed the most to prognosis, followed by N stage, RNE and surgery (Figures 1A and 2A). Various methods were then performed to identify the discriminating superiority of the nomogram. The C-indexes of the nomogram for the prediction of OS were 0.723 (95%CI, 0.711-0.735), 0.730 (95%CI, 0.714-0.747) and 0.716 (95%CI, 0.644-0.789) in the training, verification and external verification group, respectively (Table 5). The calibration curves showed no obvious deviations from the reference line, which displayed an optimal agreement between actual observations and model prediction for 3-, 5-, 10-year OS (Figure 1B, 1E and 1H). The 3-, 5-, and 10-year AUC values of the nomogram for OS were 75.98%, 73.63%, 70.45% in the training cohort (Figure 1C); 76.53%, 75.33%, 70.06% in the verification cohort (Figure 1F); and 74.74%, 78.41%, 75.62% in the external verification group (Figure 1I), which revealed an excellent sensitivity and specificity for the predictive model. Moreover, DCA demonstrated the excellent clinical utility of the comprehensive nomogram, which possessed superior net benefits to the single independent predictor (Figure 1D, 1G and 1J).

**Table 5:** The C-indices for predictions of overall survival and cancer-specific survival.

	**OS**		**CSS**	
	**C-index**	**95% CI**	**C-index**	**95% CI**
Training group	0.723	0.711-0.735	0.751	0.738-0.764
Validation group	0.730	0.714-0.747	0.755	0.738-0.773
External verification	0.716	0.644-0.789	0.712	0.638-0.785

Abbreviations: OS: overall survival; CSS: cancer-specific survival; C-index: index of concordance; CI: confidence interval.

The discriminating superiority of the nomogram predicting CSS also performed well (C-index: 0.751, 95%CI 0.738-0.764 in the training group; 0.755, 95%CI 0.738-0.773 in the verification group; 0.712, 95%CI 0.638-0.785 in the external verification group. The 3-, 5-, and 10-year AUC values: 78.74%, 76.21%, 73.19% in the training group; 78.99%, 77.28%, 72.97% in the verification group; 73.49%, 77.90%, 73.60% in the external verification group) (Figure 2).

### Performance of the nomograms in stratifying on the basis of risk points

**Figure 3:**
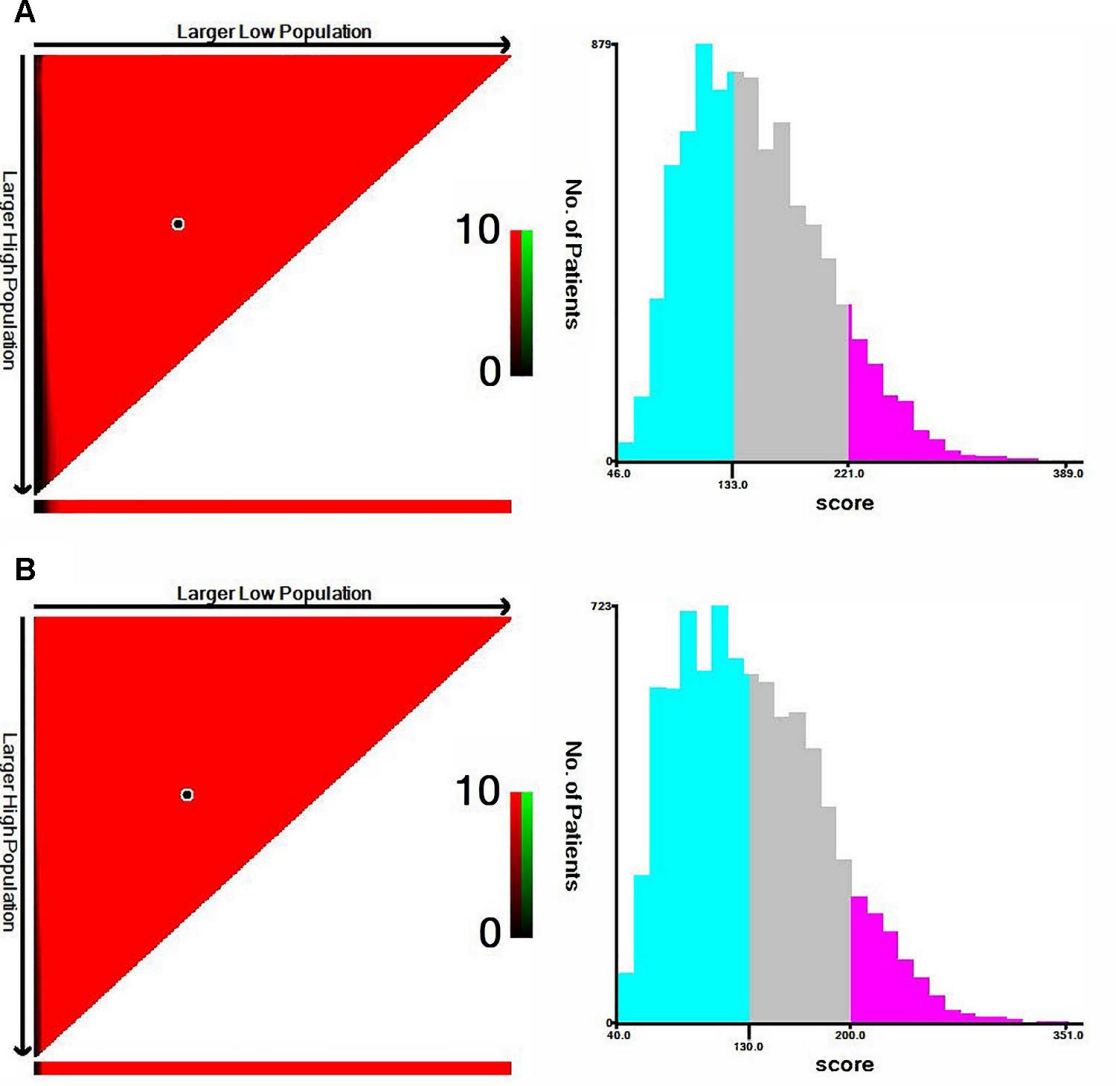
**The cut-off values were calculated by using X-tile based on the total scores of nomograms.** (**A**) The cut-off values were 133 and 221 for OS. (**B**) The cut-off values were 130 and 200 for CSS.

**Figure 4:**
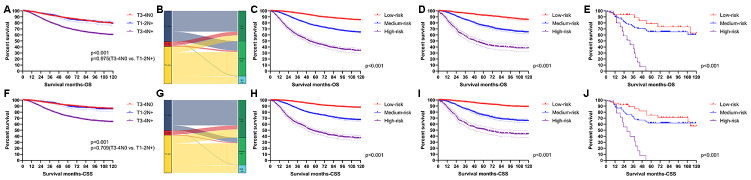
**Performance of the nomograms in stratifying on the basis of risk points.** (**A**) The difference of OS among T3-4N0, T1-2N+ and T3-4N+. (**B**) The correspondence between AJCC stage and the risk stratification based on the nomogram predicting OS. (**C**) OS in the subgroups according to the risk stratifying in the training cohort. (**D**) OS in the subgroups according to the risk stratifying in the verification cohort. (**E**) OS in the subgroups according to the risk stratifying in the external verification cohort. (**F**) The difference of CSS among T3-4N0, T1-2N+ and T3-4N+. (**G**) The correspondence between AJCC stage and the risk stratification based on the nomogram predicting CSS. (**H**) CSS in the subgroups according to the risk stratifying in the training cohort. (**I**) CSS in the subgroups according to the risk stratifying in the verification cohort. (**J**) OS in the subgroups according to the risk stratifying in the external verification cohort.

X-tile software was utilized to classify patients as high-risk, medium-risk, and low-risk, according to the risk scores of the nomograms. The cut-off values were 133 and 221 for OS (Figure 3A), 130 and 200 for CSS (Figure 3B). The survival curves in the survival paradox of the AJCC staging system for colon cancer display that patients with T3-4N0 had a similar survival to those with T1-2N+ (OS: p=0.975, Figure 4A; CSS: p=0.709, Figure 4F). Figure 4B and 4G show the correspondence between AJCC stage and the risk stratification in this study.

The risk stratification effectively avoided the survival paradox in this study. In the training cohort, the low-risk group showed the best 5-year survival rate (OS: 91.23%; CSS: 92.77%), followed by the medium-risk group (OS: 75.22%; CSS: 76.82%) and the high-risk group (OS: 46.05%; CSS: 47.71%) (Figure 4C and 4H). Similarly, the line of 5-year survival rate went from the low-risk group (OS: 92.38%; CSS: 93.59%) down to the medium-risk group (OS: 75.33%; CSS: 74.75%) and finally to the high-risk group (OS: 45.24%; CSS: 51.29%) (Figure 4D and 4I) in the verification cohort. The risk stratification system was also applicable to patients from our institution (OS: 78.79% in the low-risk group; 65.18% in the medium-risk group; 0.00% in the high-risk group; CSS: 74.90% in the low-risk group; 61.89% in the medium-risk group; 0.00% in the high-risk group) (Figure 4E and 4J).

## DISCUSSION

It is well-established that the vast majority of colon cancer occurs in patients over 50 years old. Colon cancer screening, therefore, begins in an average-risk population aged ≥50 years old [14–16]. Meanwhile, numerous studies have focused on colon cancer as a whole or even on elderly patients with colon cancer resulting in the fact that the current treatment strategies are tailored for late-onset colon cancer (in patients >50-years-old). However, early-onset colon cancer is epidemiologically, pathologically, biologically and metabolically different from late-onset colon cancer [17]. There is a current clinical unmet need regarding the diagnostic and therapeutic protocols that should be dedicated to young individuals with colon cancer.

Although widely used to evaluate the prognosis of various tumors, the AJCC staging system contains a survival paradox for locally advanced colon cancer, in that colon cancer patients with T3-4N0 (stage II) possess a similar or even worse survival compared to those with T1-2N+ (stage III) [11–13]. The survival paradox confirms that the AJCC staging system is inaccurate and insufficient for the medical demands related to locally advanced colon cancer. In fact, the root cause of the survival paradox is that T stage contributes more to prognosis than N stage, as the nomograms show. The risk stratification based on the points of the nomograms effectively avoids the survival paradox. Besides, the time-dependent ROC curve clearly shows that the nomograms possess superior sensitivity and specificity. The DCA curves indicate the comprehensive nomograms are conducive to making better clinical decisions in individual treatment compared to each independent predictor. Therefore, the survival nomograms for locally advanced colon cancer patients younger than 50years based on the SEER database are able to accurately evaluate OS and CSS of EOLACC patients and effectively solve the issue of the survival paradox.

Radical resection is the first-choice treatment for locally advanced colon cancer [18, 19]. Both nomograms predicting OS and CSS indicated the tremendous survival advantage of colectomy. Meanwhile, RNE was considered as the priority for the assessment of the quality of surgery [18, 20]. In fact, previous research identified RNE as an important prognostic factor [21, 22]. The general consensus exists that the postoperative specimens of radical operations for colon cancer should contain at least 12 regional lymph nodes, as recommended by the National Comprehensive Cancer Network (NCCN) guidelines [2]. However, previous research indicated that young patients with colorectal cancer suffered a higher risk of lymph node metastasis [23]. Is a minimum of 12 RNE adequate for EOLACC? The nomograms demonstrated that 30-35 RNE was the optimal option. Therefore, expanding lymph node dissection may be a more reasonable option for EOLACC patients.

Early-onset colon cancer patients were 2 to 4 times more likely to receive systemic chemotherapy, especially in multiagent irinotecan-based or oxaliplatin-based regimens, than late-onset patients in each disease stage [24]. However, the more intense chemotherapy did not provide young individuals with survival benefits comparable to those in late-onset colon cancer [24]. The mismatch between tumor treatment management and relative survival highlights the possibility of overtreatment and the increased risk of chemotherapy-related toxicity for early-onset colon cancer patients. Similarly, Manjelievskaia believed that the addition of systemic chemotherapy cannot offer the same survival improvement for early-onset colon cancer [4]. The nomograms confirmed that chemotherapy, which played an independent prognostic factor, contributed very little to improve OS and CSS of EOLACC in this study. Therefore, avoiding excessive chemotherapy for young colon cancer patients is the most notable finding. Patients with EOLACC were classified as high-risk, medium-risk, or low-risk according to the risk score of the nomograms in our study, which could provide a reference for EOLACC patients with respect to receiving chemotherapy or not.

Can the early-onset patients (< 50 years old) with locally advanced colon cancer be analyzed as a whole? This study divided the entire cohort into three sub-group according to age, including 18-29 years old, 30-39 years old and 40-49 years old. There is no significant difference in OS or CSS among the three subgroups in the COX regression analysis. Therefore, this study believed that it was reasonable to classify early-onset colon cancer as a whole, as many studies have done [4, 6, 24–26]. A large body of studies reported that the survival of colon cancer was related to the primary tumor location [27]. However, the primary tumor location cannot be used as an independent prognostic factor in EOLACC patients. The current treatment strategies, including surgery and chemotherapy, may bring approximate survival benefits for right colon cancer and left colon cancer.

To the best of our knowledge, this study was the first to create and validate survival nomograms for EOLACC based on the SEER database. The previous nomograms [19, 21, 28], mainly addressing elderly patients, are not suitable for early-onset colon cancer patients owing to the unequal contribution of each prognostic factor, especially chemotherapy and RNE. Our nomograms focused on EOLACC and were verified by the external information. However, there were some limitations in our study. Firstly, as a retrospective study, the nomograms still need to be validated in the future by prospective studies. Secondarily, detailed treatment information for included patients were not recorded in the SEER cohort, and we could not investigate specific options, including chemotherapy regimens and specific surgical methods, etc., in the survival of EOLACC patients. Lastly, the nomograms need to be verified by more data since the sample size of the external verification group was small.

In conclusion, we established and validated nomograms for predicting OS and CSS based on a national cohort of almost 13,000 patients with EOLACC. The nomograms could effectively solve the survival paradox of the AJCC staging system and be an excellent tool to integrate clinical characteristics to guide the therapeutic choice for EOLACC patients.

## MATERIALS AND METHODS

### Data sources

**Figure 5:**
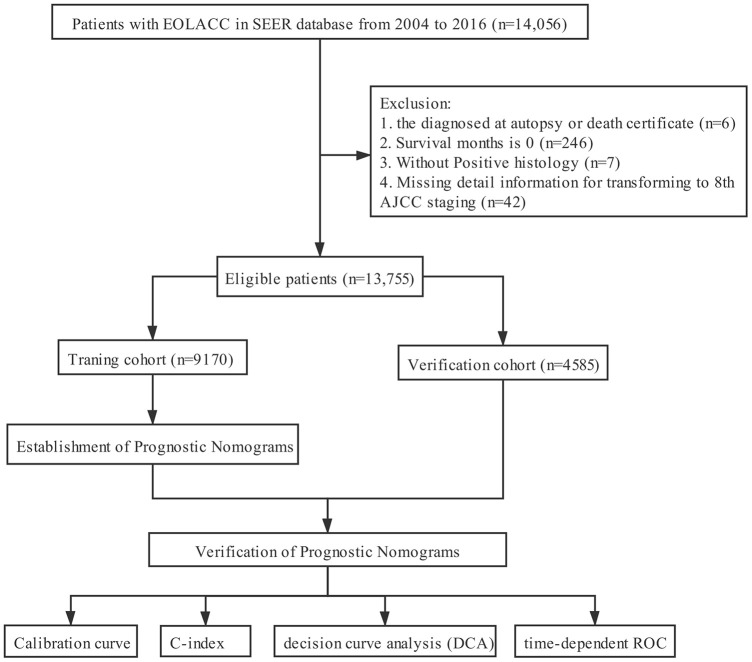
**The flow diagram.**

The clinicopathological data of all EOLACC patients were retrieved from the SEER program. The SEER Program of the National Cancer Institute is an authoritative source of information on cancer incidence and survival in the United States that is updated annually. The target population was limited to patients who were older than 18 and younger than 50, with Stage II and III colon adenocarcinoma (ICD-O-3: 8140, 8144, 8201, 8210, 8211, 8220, 8221, 8255, 8260, 8261, 8262, 8263, 8323, 8440, 8460, 8470, 8472, 8480, 8481, 8490), 14,056 patients in total. According to CS extension (http://web2.facs.org/cstage0205/colon/Colon_bao.html), T stage was re-classified to align with the 8^th^ AJCC staging system. Exclusion criteria: diagnosed at autopsy or death certificate (n=6); survival months is 0 (n=246); without Positive histology (n=7); missing detail information for transforming to 8^th^ AJCC staging (n=42). The final study sample contained 13,755 patients with early-onset locally advanced colon cancer (T3-4 and/or N+) (Figure 5).

For each patient, the following demographic, clinical, pathological and therapeutic variables were acquired: gender, age at diagnosis, race/ethnicity, marital status, tumor size, tumor location, pathological grade, histological type, T stage, N stage, surgery, chemotherapy, radiotherapy, regional nodes examined (RNE), CEA and follow-up information. All qualified patients were randomly divided into two cohorts at the ratio of 2:1 (training cohort, n =9170, and validation cohort, n =4585).

126 EOLACC patients from the Department of Gastrointestinal Surgery of Xiangya Hospital, Central South University (Changsha, China) served as the external verification group. The admission time of these patients was from January 1, 2009 to July 31, 2019. The termination of follow-up was July 31, 2020, in this study. Patients with missing follow-up data were excluded.

### Statistical analysis

A 95% confidence interval (CI) and a hazard ratio (HR) were calculated by Cox regression models. The potential prognostic factors with significant differences in the univariate Cox regression analysis were incorporated into multivariate analysis. Then, nomograms were constructed and assessed to predict 3-, 5-, and 10-year survival rates, including OS and CSS, in EOLACC patients by means of R software based on the multivariate analysis results. The distinguishing ability of the novel nomograms was verified by various methods, involving the concordance index (C-index), time-dependent receiver operating characteristic (ROC) curve and the value of the area under the ROC curve (AUC). The calibration curves were plotted to compare the nomogram-predicted survival with the actual survival. The decision curve analysis (DCA) was performed to determine the clinical usefulness by quantifying the net benefits at different threshold probabilities.

X-tile software (Yale University, New Haven, CT, USA) (version 3.6.1) was used to identify the optimal cut-off values. Statistical analyses were performed with R software (version 3.6.1, http://www.r-project.org/) and IBM SPSS software (version 25.0) (IBM, Armonk, NY, USA). The related R packages ‘rms’, ‘survival’, ‘magick’, ‘timeROC’, ‘ggplotify’ and ‘cowplot’ were applied in construction and assessment of the nomograms. All reported p-values lower than 0.05 were considered significant.

### Ethics approval

Approval from the ethical board for this study was not required because of the public nature of all the data.
